# Increased glutamine anabolism sensitizes non-small cell lung cancer to gefitinib treatment

**DOI:** 10.1038/s41420-018-0086-x

**Published:** 2018-08-09

**Authors:** Liang Wang, Wen Peng, Tianming Wu, Pengchi Deng, Ying-Lan Zhao

**Affiliations:** 1 State Key Laboratory of Biotherapy and Cancer Center, West China Hospital, West China Medical School, and Collaborative Innovation Center for Biotherapy, Sichuan University, 17#, 3rd Section, Renmin South Road, Chengdu, 610041 China; 2 Institute of Biotechnology, University of Helsinki, P.O. Box 56, 00014 Helsinki, Finland; 3 Department of Oncology, The People’s Hospital of Guizhou Province, 83#, Zhong Shan East Road, Guiyang, 550004 China; 4 Analytical & Testing Center, Sichuan University, Chengdu, 610041 China

**Keywords:** Cancer therapeutic resistance, Target identification

## Abstract

To better understand the resistance mechanism of non-small cell lung cancers (NSCLCs) to gefitinib, the metabolic profiles of gefitinib-resistant A549 cells and gefitinib-sensitive PC-9 cells were analyzed with a metabolomics analytical platform. A549 and PC-9 cells exhibited significant differences in the levels of glutamine-related metabolites. After gefitinib treatment, the glutamine level decreased in A549 cells but showed no change in PC-9 cells. The glutamine consumed by A549 cells was used to generate ATP and glutathione (GSH). As glutamine utilization was suppressed in gefitinib-treated PC-9 cells, the resulting ATP shortage and ROS accumulation led to cell death. The difference in glutamine metabolism was caused by differential changes in the levels of glutamine synthetase (GS, encoded by glutamate-ammonia ligase (*GLUL*)). *GLUL* expression was upregulated in gefitinib-sensitive cells, but it was either absent from gefitinib-resistant cells or no significant change was observed in the gefitinib-treated cells. *GLUL* overexpression in A549 cells significant sensitized them to gefitinib and decreased their invasive capacity. Conversely, knockout GS in PC-9 cells reduced gefitinib sensitivity and enhanced metastasis. Furthermore, the continuous exposure of gefitinib-sensitive HCC827 cells to gefitinib created gefitinib-resistant (GR) HCC827 cells, which exhibited a *GLUL* deletion and resistance to gefitinib. Thus, *GLUL* plays a vital role in determining the sensitivity of NSCLCs to gefitinib. Elevated GS levels mediate increased glutamine anabolism, and this novel mechanism sensitizes NSCLCs to gefitinib. The inhibition of glutamine utilization may serve as a potential therapeutic strategy to overcome gefitinib resistance in the clinic.

## Facts

1. Gefitinib-resistant and gefitinib-sensitive cells have significant differences in glutamine-related metabolism when treatment with gefitinib.
2. Gefitinib-resistant cells could escape from gefitinib-induced cell death in dependent on the glutamine metabolism, but not gefitinib-sensitive cells.
3. The differential changes in the levels of glutamine synthetase causes different glutamine metabolism between gefitinib-resistant and gefitinib-sensitive cells when treatment with gefitinib.
4. Not only in gefitinib sensitivity, the expression level of glutamine synthetase also play a role in metastasis.

## Introduction

Gefitinib is an inhibitor of epidermal growth factor receptor (EGFR) kinase, which was approved as a first-line treatment for NSCLC in 2015, however, only 10% of patients benefit from it^1^. Many factors, such as gender, smoking history, histology, and the expression and mutation of the EGFR protein, affect the sensitivity of NSCLCs to gefitinib^2,3^. Despite extraordinary progress in the clinic, the majority of gefitinib resistance mechanisms have been elucidated only by measuring altered gene or protein levels. Therefore, the application of other methods such as metabolomics to discover potential gefitinib resistance mechanisms is highly justified.

Using a combination of quantitative or flux-based metabolic approaches, and other analytical techniques, metabolic changes have been traced to alterations in enzyme kinetics^4^. The upregulation of the AKT/phosphatidylinositol 3-kinase/mammalian target of rapamycin (mTOR) signal transduction pathway activates hexokinase II activity, which redirects mitochondrial ATP to phosphorylate glucose and drives glycolysis^5^. In cancer cells, the increased dependency on glycolysis is a characteristic of multidrug-resistant (MDR) cancers and is associated with reduced sensitivity to common anticancer agents. The inhibitor of hexokinase II 3-bromopyruvate (3-BrPA) effectively inhibits glycolysis and induces cell death. Importantly, cells with the MDR phenotype remain sensitive to glycolysis inhibitors^6^. Glycolysis inhibition is an effective strategy to induce cancer cell death and overcome drug resistance. Therefore, by tracing metabolic changes, metabolomics approaches are widely used in discovering resistant mechanisms of drugs, providing new insights into pharmacodynamic properties, and elucidating the mechanisms responsible for individual variations in drug response^5,7^.

Glucose and glutamine are two primary carbon sources for energy homeostasis and biosynthesis in mammalian cells. To satisfy their requirements for energy and biosynthetic precursors, cancer cells reprogram metabolic pathways to ingest and metabolize glucose and glutamine to a degree that far exceeds their needs. Notably, to fuel abnormal cell growth and proliferation, glucose and glutamine are separately catabolized by aerobic glycolysis and glutaminolysis, which are the core hallmarks of cancer^8^. Some cancer cells increase the glutaminase (GLS) levels, which catalyzes the transformation of glutamine to glutamate, and become addicted to glutamine^9^. GLS inhibition with BPTES (*N*,*N′*-[thiobis(2,1-ethanediyl-1,3,4-thiadiazole-5,2-diyl)]bisbenzeneacetamide) under hypoxic and glucose-deficient conditions not only effectively inhibited cell proliferation in vitro but also delayed tumor xenograft growth in vivo^10^. Intriguingly, the effector of necroptosis, kinase receptor interacting protein 3 (RIP3), directly interacts with glutamine synthetase (GS), which catalyzes the reverse reaction of GLS, and glutamate dehydrogenase 1 (GLUD1) to promote RIP3-mediated necroptosis^11^.

The aim of our study is to discover novel mechanisms of NSCLC resistance to gefitinib. After exposure to gefitinib, the glutamine-related metabolic profiles showed significant differences between gefitinib-sensitive and gefitinib-resistant cells. Gefitinib-sensitive cells increased GS expression, which suppressed glutamate utilization when exposed to gefitinib. While the absence or lack of change in GS expression protects gefitinib-resistant cells from gefitinib-induced cell stress and death. We propose that the increased glutamine anabolism sensitizes NSCLCs to gefitinib treatment. The suppression of glutamine metabolism is a potential strategy to overcome the resistance of NSCLCs to gefitinib.

## Results

### ^1^H-nuclear magnetic resonance spectra reveal distinct metabolic profiles between gefitinib-treated A549 and PC-9 cells

EGFR-mutant PC-9 cells (del E746-A750) and EGFR-wild-type A549 cells were selected as representative gefitinib-sensitive and gefitinib-resistant NSCLCs, respectively. Gefitinib-induced growth inhibition was assessed using the 3-(4,5-dimethylthiazol-2-yl)−2,5-diphenyltetrazolium bromide (MTT) assay. Consistent with a previous report^12^, after treatment with gefitinib for 72 h, half maximal inhibitory concentration (IC_50_) values of 12.1 nM and 12.21 µM were observed in PC-9 cells and A549 cells, respectively. Metabolic profile was analyzed when there were no obvious cell morphology changes, which are later than metabolic changes^13^. After separately incubating A549 and PC-9 cells with 20 µM and 20 nM gefitinib for 24 h, few cells died, indicating no obvious cellular changes (Supplementary Figure S1). Therefore, the metabolic profiling analysis was performed under the same conditions.

**Fig. 1:**
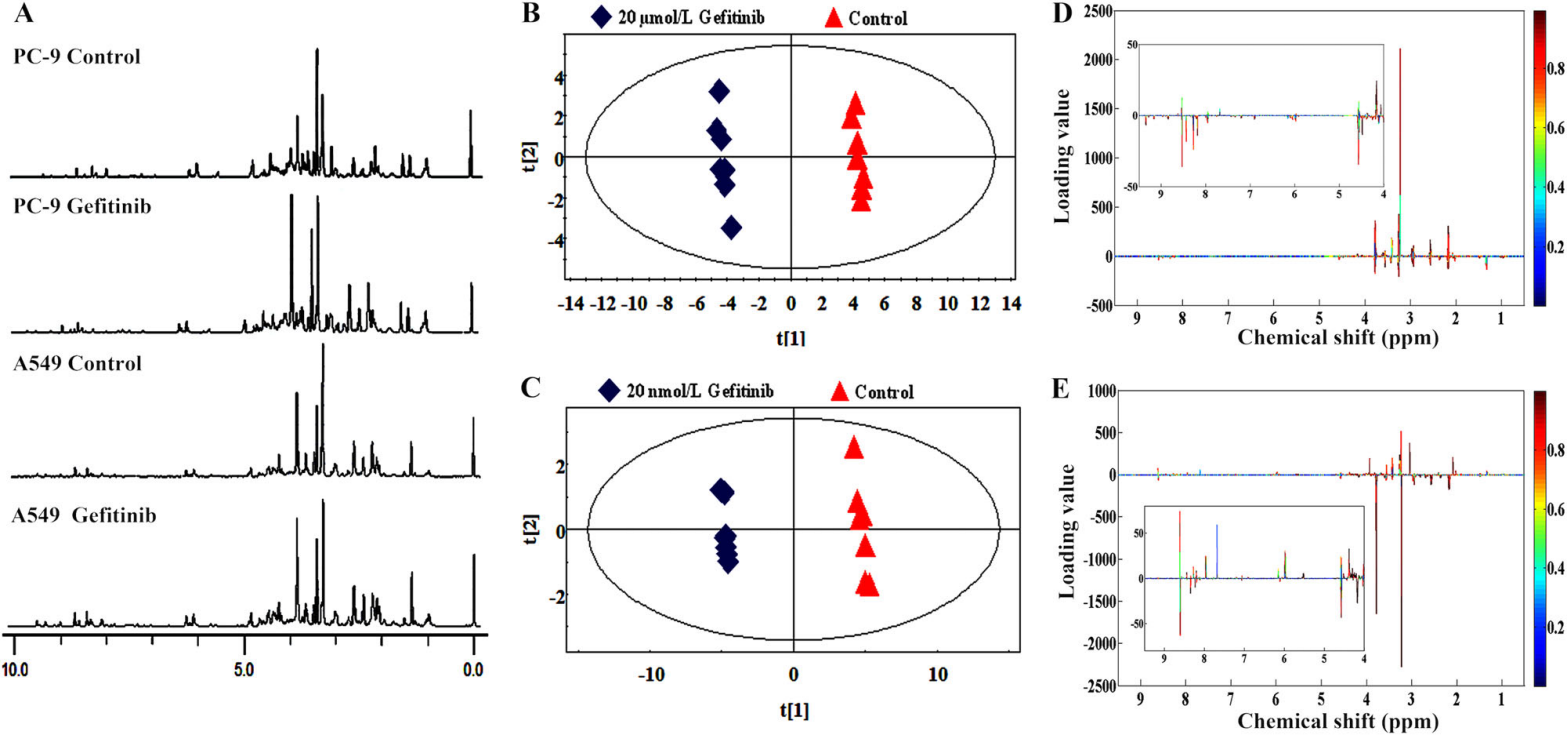
Gefitinib treatment induces differences in the metabolic profiles of PC-9 and A549 cells. **a** Representative ^1^H-NMR spectra (*δ*0.0–*δ*10.0) of PC-9 and A549 cells after a 24-h incubation with or without 20 µM and 20 nM gefitinib, respectively. **b**, **c** PCA score plots of PC1 versus PC2. The untreated A549 cells (**b**) and PC-9 cells (**c**) are shown as red triangles, and gefitinib-treated samples are shown as blue diamonds. Each group contains seven replicates. **d**, **e** The color map separately shows the significant variations in the chemical shifts of A549 cells (**d**) and PC-9 cells (**e**). Peaks in the positive direction represent enhanced chemical shifts in the gefitinib-treated group compared to the corresponding untreated group. Decreased chemical shifts in the gefitinib-treated group are presented as peaks in the negative direction

After a 24 h exposure to gefitinib, the intracellular metabolites were assessed using one-dimensional (1D) proton nuclear magnetic resonance (^1^H-NMR) spectroscopy and the representative ^1^H-NMR spectra are shown in Fig. 1a. To investigate the specific metabolic patterns, the complete set of NMR spectra was analyzed using principal component analysis (PCA), an unsupervised test that represents each individual spectrum as a single point in a score plot. The control and gefitinib-treated groups of both cell lines showed clear clustering in the PCA score plots (Fig. 1b, c). Loading plots were generated from projection to latent structures discriminant analysis (PLS-DA) to visualize the spectral variables that contributed to the separation of samples on the score plots. The loadings were colored according to variable weight and showed significant class-discriminating metabolites responsible for the clustering patterns. According to the loading plot of the principal component (PC1), several spectral areas (*δ*1.3, *δ*2.0, *δ*2.1, *δ*2.5, *δ*2.9, *δ*3.0, *δ*3.2, *δ*3.4, *δ*3.5, *δ*3.7, and *δ*3.9) of A549 cells (Fig. 1d) were significantly different from the PC-9 cells (Fig. 1e). The changes in these spectral areas not only caused the separation between control and gefitinib-treated groups but also implied differences in metabolic reprogramming between A549 and PC-9 cells. Together, the gefitinib treatment causes profoundly different metabolic profiles between two cell lines.

### Glutamine-related metabolism may affect the sensitivity of NSCLCs to gefitinib

**Fig. 2:**
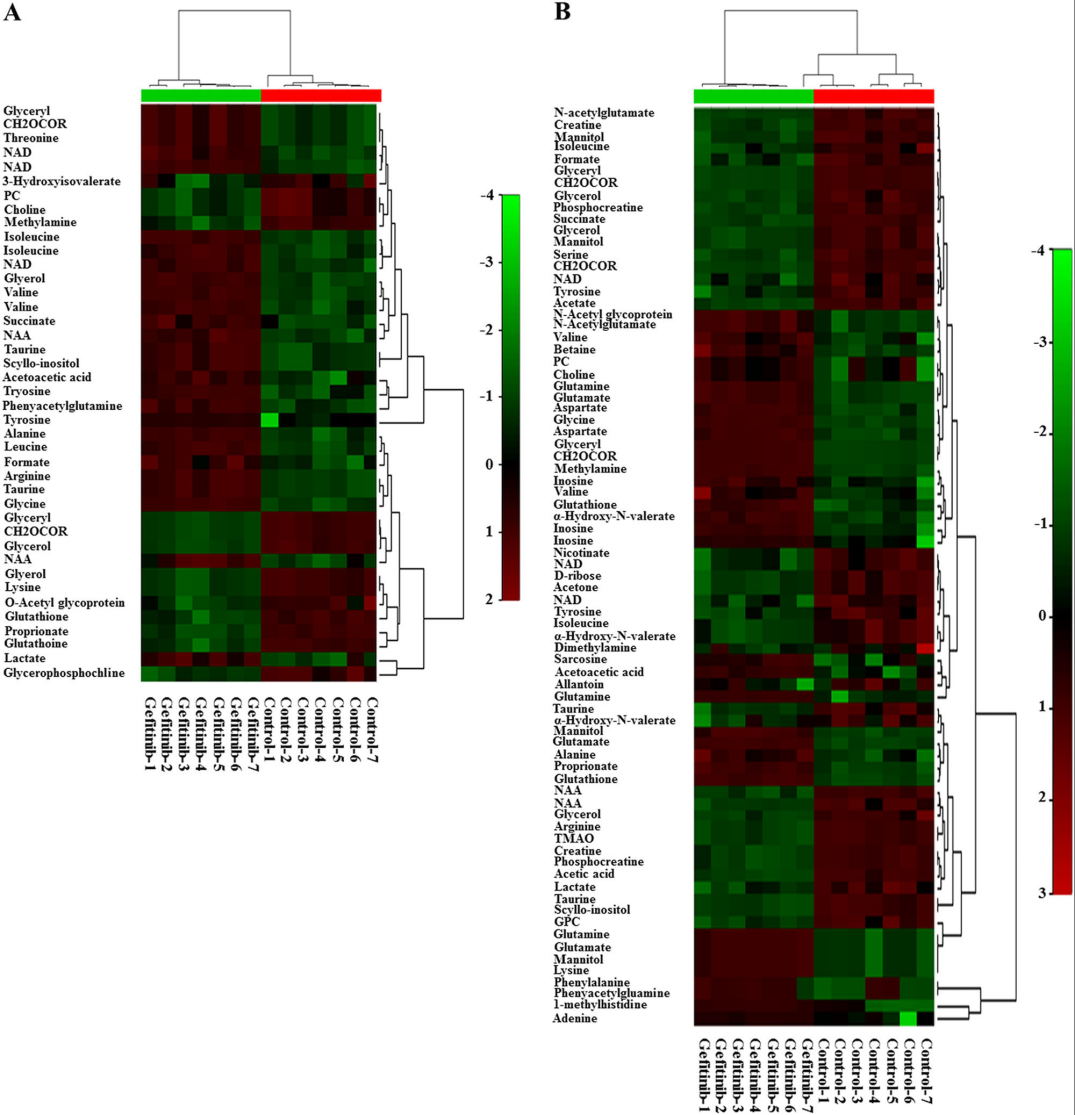
Comparative metabolomics indicates that glutamine-related metabolism affects the sensitivity of NSCLCs to gefitinib. **a**, **b** Heat map representation of a 2D hierarchical clustering of metabolites identified as differentially changed in A549 cells (**a**) and PC-9 cells (**b**) after treatment with gefitinib compared to control cells. Each column represents a treatment group, and each row represents a metabolite

A color scale, corresponding to the Par model variable from the loading plots, was used to identify the significant class-discriminating metabolites involved in the clustering patterns. The corresponding metabolites exhibiting significant changes in their chemical shifts compared to the reference standards (variable importance in the projection (VIP) value > 1 and *p* < 0.05) were summarized in Supplementary Table S1 and S2. As shown in tables, 29 and 51 metabolites were separately identified in A549 and PC-9 cells, which showed significant change in response to the gefitinib treatment. To visualize the similarities and relationships in the metabolites, the two-dimensional hierarchical clustering of seven pairwise samples, and the corresponding discriminating metabolites, are presented in heat map (Fig. 2a, b). Except for one gefitinib-treated sample, which was clustered into control group in PC-9 cells (Fig. 2b), all other samples were correctly clustered into corresponding control and gefitinib-treated groups. Interestingly, all discriminating metabolites in A549 cells can be observed in PC-9 cells. While the remaining 22 metabolites only exhibited specific changes in PC-9 cells. Among these metabolites, glutamine and glutamate are directly involved in glutamine metabolism. Aspartate^14^ and *N*-acetylglutamate^15^ are correlated with the glutamine metabolism-related urea cycle. Phosphocreatine and creatine could compensate for the lack of ATP synthesis^16^, while acetic acid and acetone are the final product of the bypass pathway of glucose metabolism^17^, and final product of glycolysis, respectively. The end product of xanthine metabolism and uric acid, allantoin, is a marker of oxidative stress^18^. The specific changes of these metabolites in PC-9 cells may sensitize the cells to gefitinib, or may indicate metabolic reprogramming induced by gefitinib treatment.

Cancer cells reprogram their metabolic patterns and preferentially catabolize glucose and glutamine through aerobic glycolysis and the Krebs cycle, respectively^19^. Thus, the metabolites related to glucose and glutamine metabolism were further analyzed. Four metabolites (glutamine, glutamate, aspartate, and *N*-acetylglutamate) and two metabolites (acetic acid and acetone) were associated with glutamine and glucose metabolism, respectively. Based on the VIP and fold change values, changes in glutamine metabolism-related metabolites were more evident than glucose metabolism-related metabolites (Supplementary Table S3). Therefore, we suspect that glutamine-related metabolism plays a pivotal role in determining gefitinib sensitivity.

### Glutamine utilization was reduced in PC-9 cells but not in A549 cells in response to gefitinib

**Fig. 3:**
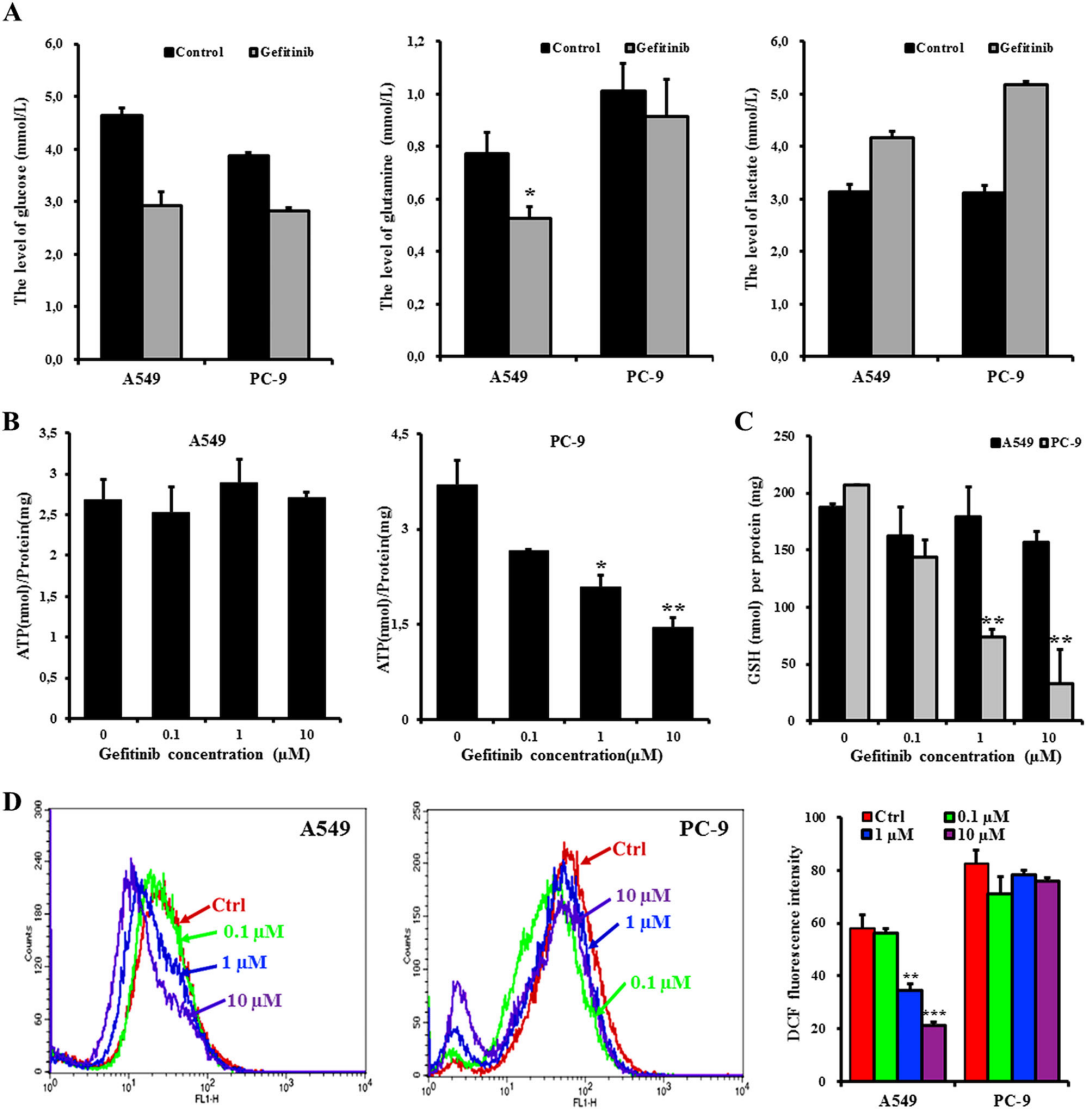
The inhibition of glutamine utilization in PC-9 cells reduces ATP and GSH generation, which induces cell death in response to gefitinib. However, A549 cells utilize glutamine to survive the gefitinib treatment. **a** Levels of glucose, glutamine, and lactate in A549 and PC-9 cells after a 24-h treatment with or without 20 µM and 20 nM gefitinib, respectively. **b**–**d** After A549 and PC-9 cells were exposed to 0, 0.1, 1, and 10 µM gefitinib for 72 h, the intracellular ATP (**b**), GSH (**c**), and ROS levels (**d**) were measured using the ATP determination kit, the GSH-Glo glutathione assay kit, and the DCFH-DA reagent, respectively. The total ATP and GSH levels were normalized to the total protein concentration that was used for ATP and GSH assays. The histogram shows the mean fluorescent intensity of DCF in the control and gefitinib-treated groups. The data represent the mean ± SEM of three independent experiments. *p *<* 0.05; **p *<* 0.01; ***p *<* 0.001, two-tailed Student’s *t*-test

To verify the role of glutamine in determining the sensitivity of NSCLCs to gefitinib, the levels of glucose, glutamine, and lactate, a final product of aerobic glycolysis, were assessed. After separately exposing A549 and PC-9 cells to 20 µM and 20 nM gefitinib for 24 h, the glucose concentration decreased and the lactate level increased in both cell lines compared to control cells. However, gefitinib treatment only decreased the glutamine levels in A549 cells, with no obvious changes in PC-9 cells (Fig. 3a). Thus, both cell lines maintain the same level of glucose consumption when treated with gefitinib, but the glutamine utilization is higher in A549 cells than in PC-9 cells. Consistent with the results from the ^1^H-NMR analysis, the glutamine-mediated metabolic pathways, but not glucose metabolism, correlate with gefitinib resistance in NSCLCs.

Next, the functions of glutamine-mediated metabolic pathways were further evaluated to identify the mechanism by which the glutamine-related metabolic pathway suppresses gefitinib sensitivity in NSCLCs. As glutamine-derived glutamate can be converted into α-ketoglutarate by GLUD1 to fuel the tricarboxylic acid (TCA) cycle in the mitochondria, which generates reductive hydrogen for the respiratory chain to produce ATP^20^. Therefore, ATP levels were analyzed to examine whether the consumed glutamine by A549 cells was used to supply ATP. As gefitinib concentrations were increased (0, 0.1, 1, and 10 µM), the ATP concentration gradually decreased in PC-9 cells, whereas were maintained stable in A549 cells (Fig. 3b).

In addition to supporting ATP production, glutamine-derived glutamate provides precursors for synthesizing GSH, an important mitochondrial ROS scavenger. Furthermore, when glutamine-derived oxaloacetate is metabolized into malate, the produced NADPH provides the reductive power to maintain the reduced GSH pools^20^. Elevated GSH levels increases the antioxidant capacity and reduce the sensitivity of many tumors to anticancer agents^21^. Thus, to determine whether the consumed glutamine was converted to GSH, both the GSH and reactive oxygen species (ROS) levels were assessed. After treatment with gefitinib for 72 h, the GSH levels in PC-9 cells was reduced in a dose-dependent manner, which induced low ability to scavenge the accumulated ROS levels (Fig. 3c, d). Unlike PC-9 cells, A549 cells utilized glutamine to maintain stable GSH levels even after treatment with 10 µM gefitinib (Fig. 3c). The stable GSH levels efficiently scavenged ROS, which was reduced in the gefitinib-treated cells (1 and 10 µM) when compared to control cells (Fig. 3d). Thus, the utilized glutamine by A549 cells was catabolized to provide ATP, and to synthesize GSH that protects the gefitinib-resistant cells from ROS-induced damage. However, gefitinib-sensitive cells cannot metabolize glutamine to circumvent the gefitinib-induced metabolic stress.

### GS inhibits glutamine metabolism in gefitinib-sensitive cells

**Fig. 4:**
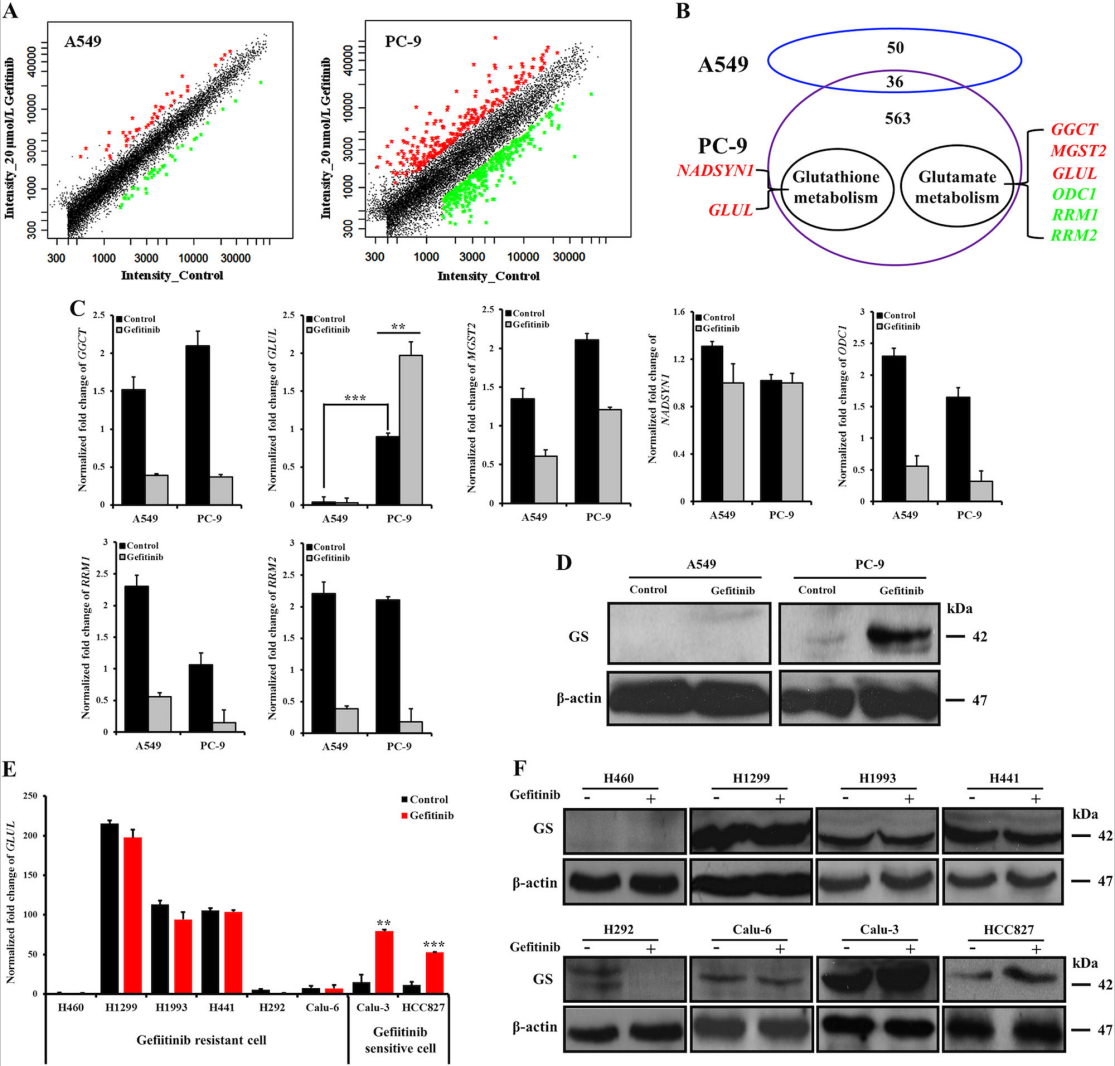
*GLUL* and GS levels were upregulated in gefitinib-sensitive cells in response to the gefitinib treatment. Gefitinib-resistant cells lack *GLUL* expression or exhibit no significant changes following the gefitinib treatment. **a** After separately exposing A549 and PC-9 cells to 20 µM and 20 nM gefitinib, respectively, for 48 h, DNA microarray scatter plots were prepared to reveal the expression of activation-induced genes in gefitinib-treated cells compared with that in the corresponding control cells. Each point represents a gene; the red points indicate genes that significantly upregulated in gefitinib-treated cells (ratio ≥ 2-fold, *p* *<* 0.05), whereas the green points indicate genes that were significantly downregulated (ratio ≤ 0.5-fold, *p* *<* 0.05) in response to the gefitinib treatment. The black points represent genes for which the signal intensity ratio was between 0.5 and 2, indicating that gefitinib treatment had no obvious effect on these genes. **b** A scheme displays the relationships between the differentially expressed genes in A549 and PC-9 cells. The genes related to glutamine metabolism are listed. The red- and green-colored genes represent increased and decreased gene expression, respectively, in gefitinib-treated cells. **c** Changes in the mRNA expression levels of seven important genes (*GGCT*, *GLUL*, *MGST2*, *NADSYN1*, *ODC1*, *RRM1*, and *RRM2*) in A549 and PC-9 cells in response to the 48-h gefitinib treatment are shown. The data represent the mean ± SEM of three independent experiments. *p < 0.05; **p < 0.01; ***p < 0.001, two-tailed Student’s *t*-test. **d** Western blot detection of the levels of the GS protein in A549 and PC-9 cells after treatment with 20 µM and 20 nM gefitinib, respectively, for 48 h. **e**, **f** Changes in *GLUL* mRNA expression levels were quantified by qRT-PCR (**e**), and the GS protein levels were examined by western blotting (**f**) in cells treated with gefitinib for 48 h and the corresponding control cells. The bars shown are normalized to the GAPDH control and represent the mean ± SD of triplicate samples

To determine which proteins are responsible for the different glutamine metabolism between sensitive and resistant cells in response to the gefitinib treatment, cDNA microarrays were used to examine the global mRNA expression levels, with a particular focus on genes involved in glutamine metabolism. As determined from the signal intensities in the scatter plots (Fig. 4a), 599 genes were differentially expressed in PC-9 cells. Among these genes, 251 genes were upregulated and are shown as red spots (ratio value ≥ 2-fold, *p* < 0.05), while 348 genes were downregulated and are shown as green points (ratio value ≤ 0.5-fold, *p* < 0.05) (Fig. 4a and Supplementary Table S4). In contrast, the gefitinib-treated A549 cells only exhibited significant changes in 86 genes compared with control cells. As shown in Fig. 4a and Supplementary Table S5, 44 and 42 genes were increased and decreased, respectively. Based on previous studies and the Kyoto Encyclopedia of Genes and Genomes (release 41.1, http://www.genome.jp/kegg) database, these genes were mapped to the corresponding metabolic pathways. Excluding genes that exhibited changes in both cell lines or related to the cell cycle, 7 genes were identified as being associated with glutamine or glutamine-related metabolic pathways. In addition to *GLUL*, which participates in both pathways, *NADSYN1* and 5 other genes (*GGCT*, *MGST2*, *ODC1*, *RRM1* and *RRM2*) were found to correlate with glutathione and glutamate metabolism (Fig. 4b).

Next, quantitative real-time PCR (qRT-PCR) further verified the changes in these genes and found six genes expressed similar in both cells, except for the *GLUL*, which was differentially expressed between A549 and PC-9 cells and exhibited a different change after gefitinib treatment (Fig. 4c). The *GLUL* expression level was much higher in PC-9 cells than in A549 cells, in which levels were nearly undetectable. Interestingly, gefitinib treatment induced a more than 20-fold increase in the *GLUL* levels in PC-9 cells, but was even slightly reduced in A549 cells. Consistent with mRNA level, gefitinib treatment also significantly boosted GS protein level in PC-9 cells, while there was no detectable GS increase in A549 cells (Fig. 4d).

Furthermore, changes in *GLUL* and GS levels were assessed in several other gefitinib-resistant NSCLC cell lines (H460, H1299, H1993, H441, H292, and Calu-6) and gefitinib-sensitive NSCLC cell lines (Calu-3 and HCC827), after treatment with equal gefitinib concentration to IC_50_ value (Supplementary Table S6) Among the gefitinib-resistant cells, except for H460 cells, which were similar to A549 cells and lack of *GLUL* and GS expression, the other five cell lines expressed *GLUL* and GS. However, gefitinib treatment did not change the *GLUL* and GS expression levels. Conversely, gefitinib treatment even mediated the absence of *GLUL* and GS expression in H292 cells. Unlike gefitinib-resistant cells, Calu-3 and HCC827 cells exhibited a significant increase in the *GLUL* and GS levels in response to gefitinib treatment (Fig. 4e, f). Thus, GS expression level is not a suitable marker to distinguish gefitinib-sensitive and gefitinib-resistant cells. However, the upregulation of GS level upon gefitinib treatment may be used to determine whether NSCLCs are sensitive to gefitinib.

### Changing the GS expression level alters the susceptibility of A549 and PC-9 cells to gefitinib

**Fig. 5:**
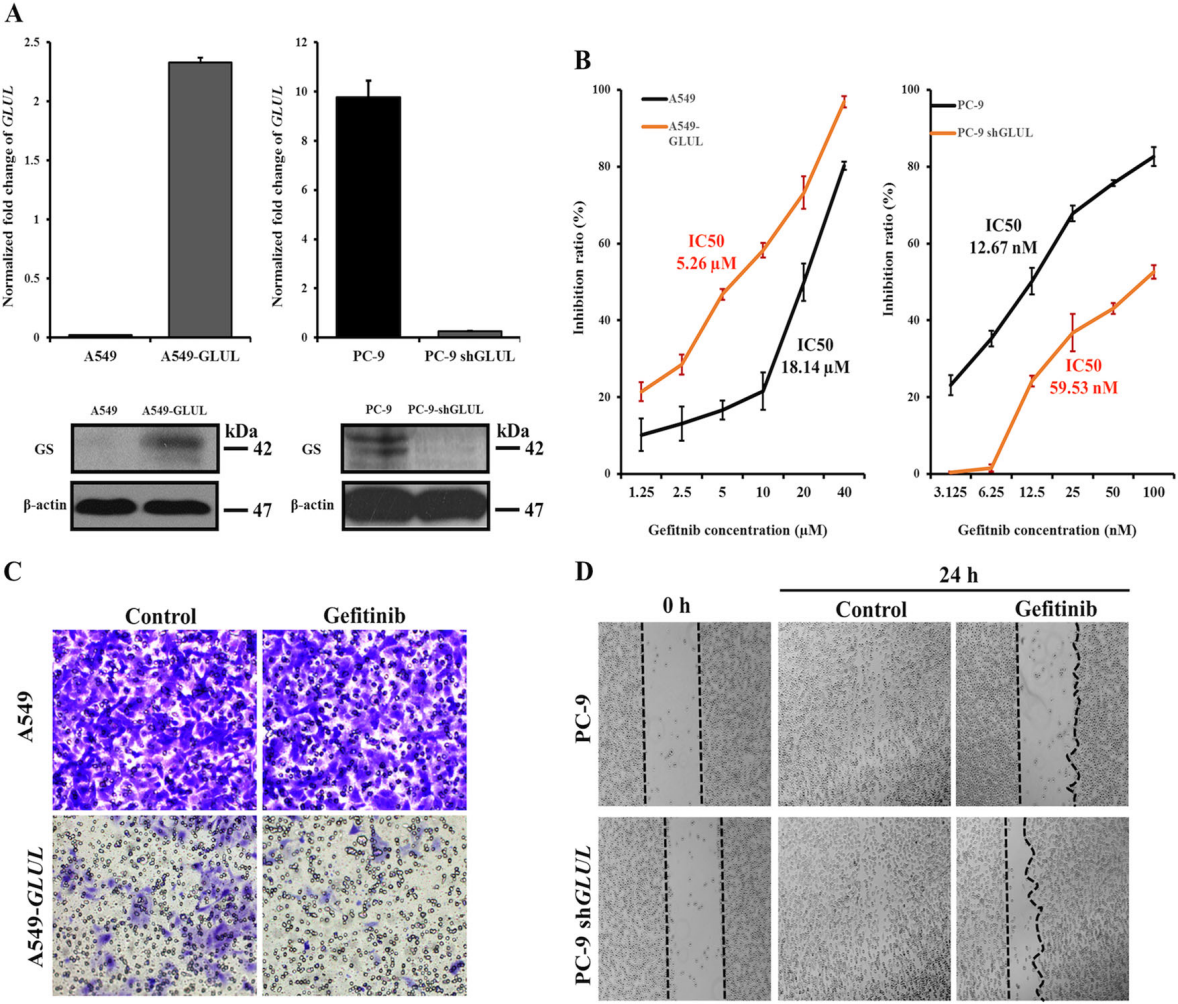
Expression of *GLUL* in A549 cells sensitizes them to the gefitinib treatment and decreases cell motility, whereas the loss of *GLUL* expression in PC-9 cells increases resistance to gefitinib treatment and increases cell motility. **a** qRT-PCR and western blotting were used to assess the *GLUL* mRNA level and the GS protein level, respectively, to identify the *GLUL* knock-in efficacy in A549 cells and the *GLUL* knockout efficacy in PC-9 cells. The bars shown are normalized to the GAPDH control and represent the mean ± SD of triplicate samples. **b** MTT assays detected the cell growth inhibition ratios following the gefitinib treatment in *GLUL*-expressing A549 cells and *GLUL* knockout PC-9 cells. **c** Based on the transwell assay, significantly fewer A549-*GLUL* cells invaded the membrane than A549 cells, and the 24-h gefitinib treatment further suppressed the invasion of A549-*GLUL* cells. In contrast to A549-*GLUL* cells, the gefitinib treatment did not inhibit the invasion of A549 cells. **d** Scratch wound-healing assays showed that the knockout of *GLUL* in PC-9 cells resulted in a decrease of the ability of cells to close a wound after the 24-h treatment with gefitinib

To test whether change GS level would alter the sensitivity of A549 and PC-9 cells to gefitinib, the lentivirus-based system was applied to knock-in GS in A549 cells (A549-*GLUL*) or to knockout endogenous GS expression in PC-9 cells (PC-9 sh*GLUL*). After analysis of *GLUL* and GS level (Fig. 5a), the sensitivity to gefitinib was evaluated by MTT assay. As shown in Fig. 5b, A549-*GLUL* cells displayed more sensitivity to the gefitinib treatment than A549 cells. The IC_50_ value decreased from 18.14 µM in A549 cells to 5.26 µM in A549-*GLUL* cells. However, the absence of *GLUL* in PC-9 cells induced less sensitivity to gefitinib and the IC_50_ value increased from 12.67 nM in PC-9 cells to 59.53 nM in PC-9 sh*GLUL* cells. Thus, changes in GS expression altered the susceptibility of NSCLCs to gefitinib.

In addition to tumorigenesis, glutamate also plays a role in increasing pancreatic cancer cell invasion and migration via AMPA receptor activation and KRAS-MAPK signaling^22^. To test whether the increased conversion of glutamate to glutamine, caused by increase of GS expression, also suppressed the migration of NSCLCs, the invasive capacity of A549 and A549-*GLUL* cells was assessed by Transwell migration assay. As shown in Fig. 5c, A549-*GLUL* cells showed decreased transmembrane invasion compared with A549 cells. Interestingly, gefitinib treatment further decreased the invasive capacity of A549-*GLUL* cells but had no apparent effect on A549 cells. Furthermore, a scratch wound-healing model was used to evaluate the migration ability of PC-9 and PC-9 sh*GLUL* cells. Although there was no significant difference in control cells, gefitinib treatment mediated longer length of scratch wound in PC-9 cells than in PC-9 sh*GLUL* (Fig. 5f). Therefore, increased GS expression not only sensitize NSCLCs to gefitinib but also play a role in decreasing the migration and invasion of NSCLCs.

### Acquired resistance of NSCLCs to gefitinib is associated with the loss of GS expression

**Fig. 6:**
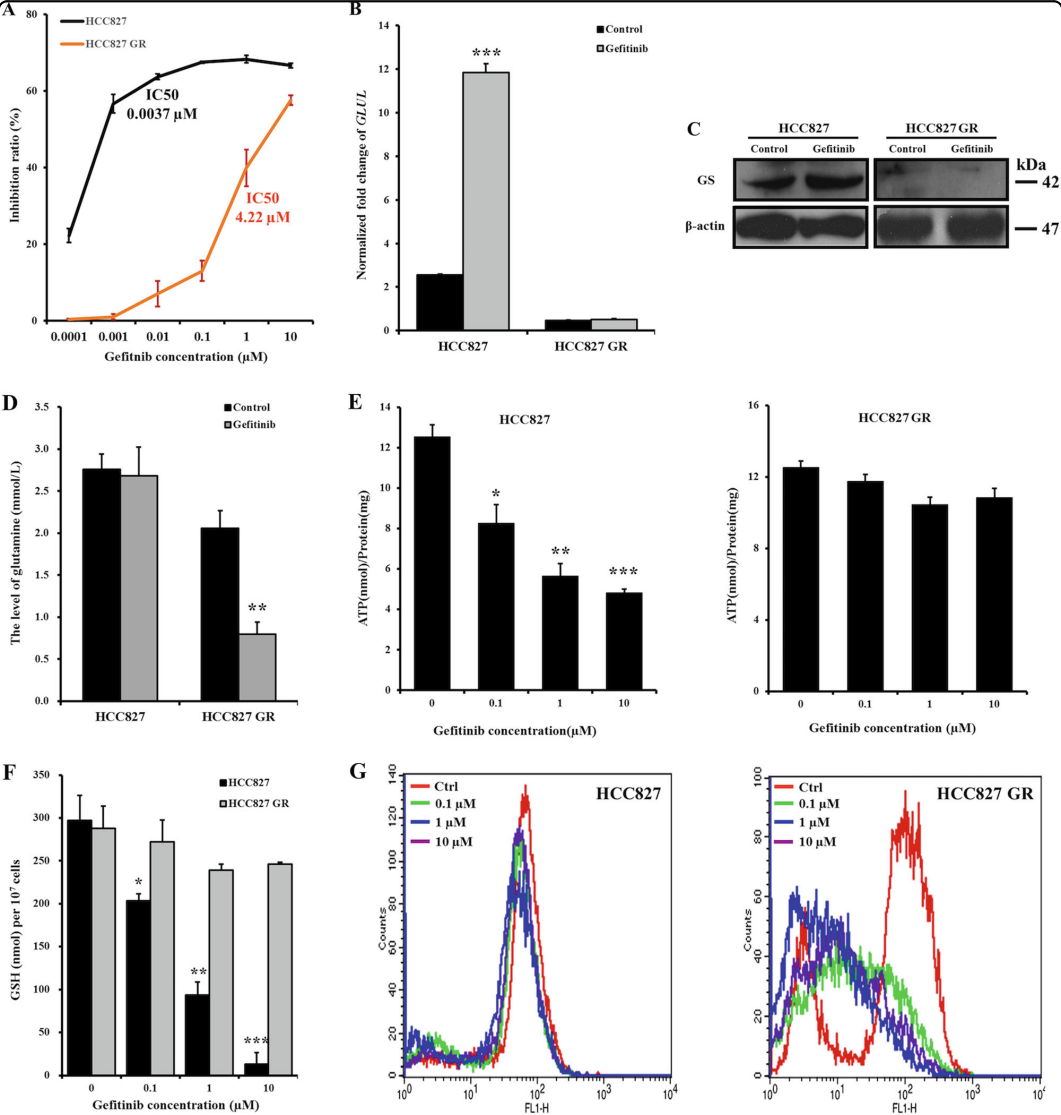
Decreased GS expression in HCC827 cells is associated with acquired resistance to gefitinib. **a** According to the MTT assays, HCC827 GR cells became resistant to gefitinib after chronic and repeated exposure to increasing doses of gefitinib compared to HCC827 cells, which were sensitive to gefitinib. **b**, **c** Changes in *GLUL* mRNA expression levels were quantified by qRT-PCR (**b**), and GS protein levels were examined by western blotting (**c**) to compare the levels between HCC827 and HCC827 GR cells after exposure to the gefitinib or control treatment for 48 h. The bars shown are normalized to the GAPDH control and represent the mean ± SD of triplicate samples. **d** Glutamine levels in HCC827 and HCC827 GR cells were assessed after a 24-h exposure to 5 µM and 5 nM gefitinib, respectively. **e**–**g** After exposing HCC827 and HCC827 GR cells to 0, 0.1, 1, and 10 µM gefitinib for 72 h, the intracellular ATP (**e**), GSH (**f**), and ROS levels (**g**) were measured using the ATP determination kit, the GSH-Glo glutathione assay kit, and the DCFH-DA reagent, respectively. The gefitinib treatment significantly reduced the normalized ATP and GSH levels in HCC827 cells in a dose-dependent manner. Compared to the stable ROS level in HCC827 cells, the total ROS level in HCC827 GR cells was reduced, indicating scavenging. The data represent the mean ± SEM of three independent experiments. *p < 0.05; **p < 0.01; ***p < 0.001, two-tailed Student’s *t*-test

Acquired resistance is another reason that leads to poor or no response to gefitinib therapy. To test whether the loss of GS expression is also involved in acquired resistance, a cell line with acquired resistance to gefitinib, HCC827 GR, was established by chronically and repeatedly exposing HCC827 cells to increasing doses of gefitinib, leading to an IC_50_ value in HCC827 GR cells of 4.22 µM. Compared to HCC827 cells with an IC_50_ value of 0.0037 µM, the sensitivity of HCC827 GR cells to gefitinib decreased more than 1000-fold (Fig. 6a). Both *GLUL* and GS levels were then evaluated and found that decreased sensitivity to gefitinib correlated with the depletion of *GLUL* and GS expression in HCC827 GR cells. Similar to PC-9 cells, gefitinib treatment also significantly increased the *GLUL* and GS levels in HCC827 cells (Fig. 6b, c).

Next, glutamine levels were analyzed to assess glutamate utilization. After treated with 5 nM gefitinib for 24 h, there was no difference compared to control HCC827 cells. However, glutamine levels significant decreased in HCC827 GR cells after treatment with 5 µM gefitinib, when compared to control cells (Fig. 6d). Due to the inhibited glutamine utilization in HCC827 cells, glutamine cannot be transformed to provide ATP and GSH, which reduced in a dose-dependent manner in response to gefitinib treatment. However, similar to A549 cells, the decreased glutamine in HCC827 GR cells was utilized to maintain ATP and GSH level, which were shown no obviously change compared to control cells in response to gefitinib treatment (Fig. 6e, f). Undoubtedly, the low level of GSH in HCC827 cells cannot scavenge the accumulated ROS in cells, which further damages cells and induces cell death. In contrast, ROS were scavenged by stable GSH levels in HCC827 GR cells, and thus cells were protected from ROS-induced oxidative stress (Fig. 6g). More data are needed to confirm whether the acquired resistance of NSCLCs to gefitinib mediated the loss of GS, or the loss of GS reduced the sensitivity of NSCLCs to gefitinib, but the absence of GS is a potential factor linked to acquired resistance. These results further support the key roles of GS and glutamine-related metabolic pathways in determining the sensitivity of NSCLCs to gefitinib.

## Discussion

**Fig. 7:**
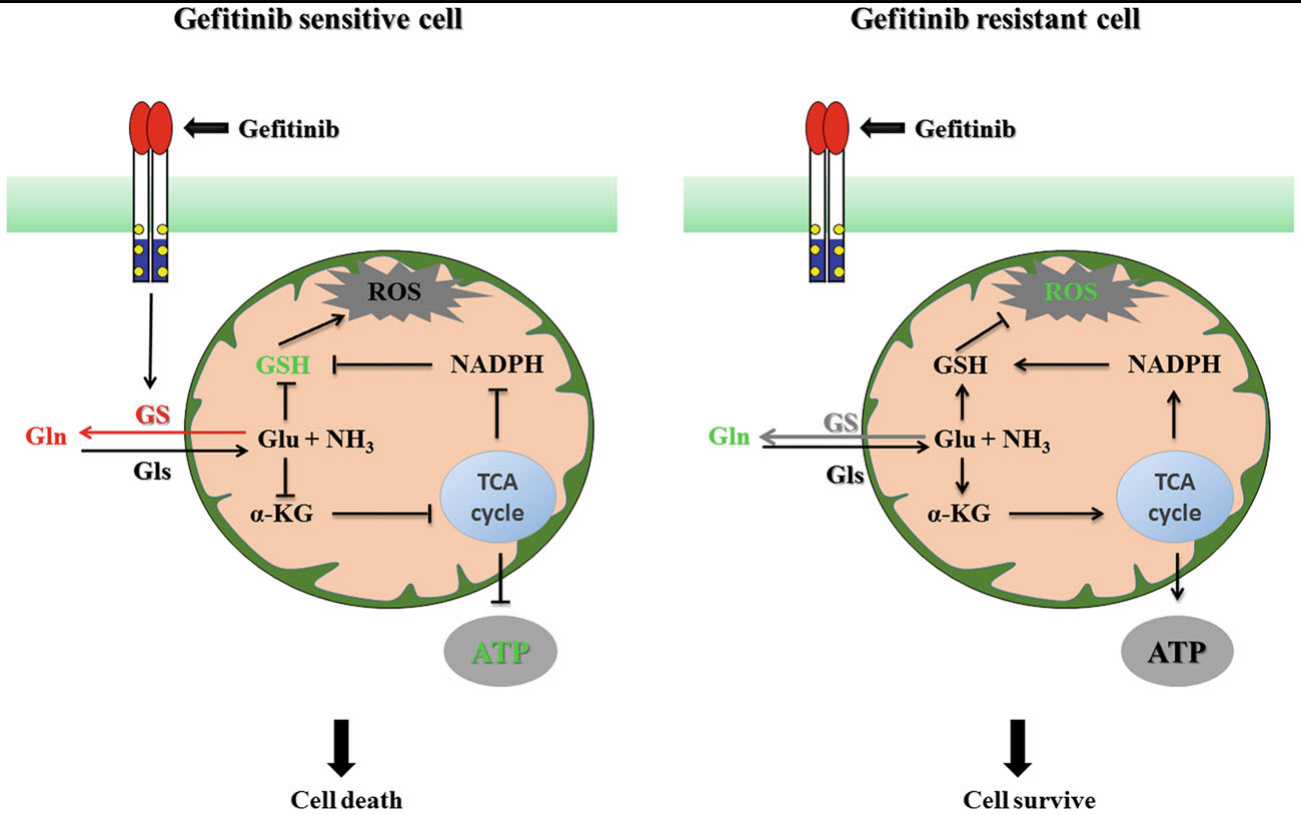
The increased glutamine anabolism promoted by GS expression sensitizes NSCLCs to gefitinib by attenuating energy production and GSH generation, leading to cell death. However, glutamine is utilized when GS is not expressed or when GS expression does not change; thus, glutamate is converted to energy and GSH to protect cells from gefitinib-induced cell stress. The GS and glutamine levels are increased in gefitinib-sensitive cells in response to the gefitinib treatment, leading to decreased GSH and ATP levels. The glutamine level is reduced in gefitinib-resistant cells, and the utilized glutamine efficiently scavenges the accumulated ROS. Taken together, the combination of gefitinib with an inhibitor of glutamine utilization, such as an inhibitor of GLS activity, is a rational therapeutic strategy to overcome gefitinib resistance in patients with NSCLC

Taken together, our studies unravel the inhibition of glutamate utilization by increased GS expression as an important signal transducer toward a more sensitive and low invasive phenotype when applying gefitinib in NSCLC therapy (Fig. 7). By analyzing the ^1^H-NMR profiles of different acute myeloid leukemia (AML) cells treated with a combination of bezafibrate and medroxyprogesterone acetate, Tiziani et al.^23^ showed that the unexpected antileukemic activities of this combination against AML were associated with ROS generation rather than an unexpected mechanism. In our study, the difference in ^1^H-NMR metabolic profiles between gefitinib-sensitive and gefitinib-resistant cells was mainly caused by glutamine-related metabolic pathways. Glutamine plays many important roles in redox homeostasis, energy formation, macromolecular synthesis, and signaling in cancer cells^24^. In the glioblastoma cells, the activation of glutamine metabolism through increased GLS activity raises glutamate level, which was utilized to fuel the TCA cycle, and finally induced resistance to mTOR inhibitor^25^.

By stimulating glutaminolysis, transformed cancer cells become addicted to glutamine to maintain robust cell proliferation. In patients with breast cancer, a higher glutamate-to-glutamine ratio is associated with prolonged overall survival^26^. The enhanced glutamine metabolic pathway is helpful for improving the survival of cancer cells. Compared to loss of ability to catabolize glutamine in gefitinib-sensitive cells, gefitinib-resistant cells still utilize glutamine in response to gefitinib (Fig. 3a). Glutamine deprivation enhances the antitumor activity of 3-BrPA in cancer cells by increasing metabolic-oxidative stress and decreasing ATP levels^27^. Our study also found that a lack of ATP and scavenge oxidative stress caused by inhibiting glutamine utilization sensitize PC-9 cells to gefitinib, while the consumed glutamine in A549 cells was metabolized to generate ATP and GSH, which satisfies energy requirement and protects cells from ROS-induced cell damage, respectively (Fig. 3b, c). Therefore, upon exposure to gefitinib, A549 cells but not PC-9 cells, survived by depending on glutamine metabolism. Furthermore, the reduced circulating glutamine levels not only significantly inhibited the proliferation of VM-M3 cells but also inhibited long-distance metastasis both in vitro and in vivo^28^. We also found that GS-expressed A549 cells exhibited decreased invasion compared to that in A549 cells. However, loss of GS decreased the migration of PC-9 cells upon treatment with gefitinib.

To establish a specific metabolic phenotype and control metabolic flux rates, some key metabolic enzymes are abnormally expressed in cancer cells^29,30^. The elevated GLS level and activity correlates with the high growth rate and the extended cell survival in some neuroblastomas^31,32^. Under the gefitinib treatment, the specifically upregulated GS level in gefitinib-sensitive cells, not in gefitinib-resistant cells, indicates that GS plays a key role in controlling different glutamine-related metabolic profiles between gefitinib-sensitive and gefitinib-resistant cell lines. GLS and GS catalyze the same reaction in opposite directions and commonly modulate intracellular glutamine/glutamate levels^33^. A high ratio of *GLS/GLUL* mRNA expression in a tumor is indicative of a dependence on extracellular glutamine for cell growth and survival^34^. By disrupting the high *GLS*/low *GLUL* mRNA expression pattern, the GLS inhibitor CB-839 induced an increase in tumor glutamine levels and a decrease in glutamate levels, ultimately causing significant tumor growth inhibition both in vitro and in vivo^35^. The *GLS/GLUL* mRNA ratio in PC-9 cells was not only much lower than the ratio in A549 cells, but further decreased in PC-9 cells after the gefitinib treatment. However, no obvious change occurred in A549 cells (Supplementary Figure S2). As the gefitinib treatment downregulated the *GLS* level in both cell lines (Supplementary Figure S3), the decreased *GLS/GLUL* ratio was mainly due to increased *GLUL* expression in PC-9 cells. The elevated level of GS catalyzes the formation of glutamine from glutamate, which inhibiting glutamate utilization in PC-9 cells. In contrast, due to the decreased *GLUL* expression in A549 cells, glutamate is metabolized to maintain normal ATP levels and to reduce ROS-induced cell damage following the gefitinib treatment (Fig. 3).

In conclusion, the different expression levels of GS between gefitinib-sensitive and -resistant cells causes an abnormality in the metabolic networks of glutamine utilization, which establishes a potentially novel mechanism underlying both primary and acquired resistance to gefitinib treatment in patients with NSCLC. These findings may have important implications for treatments combating gefitinib resistance in patients with NSCLC and suggest that a combination of gefitinib with glutamine metabolic inhibitors should be used.

## Materials and methods

### Reagents and antibodies

Deuterium oxide (99.8% D) was purchased from NORELL (Landisville, USA). Trimethylsilylpropionic acid-d4 sodium salt (TSP) was obtained from Sigma-Aldrich (St. Louis, MO). High-performance liquid chromatography-grade methanol and chloroform were purchased from Fisher Scientific (Fairlawn, NJ, USA). Deionized water was obtained from an EASYpure II UV water purification system (Barnstead International, Dubuque, IA). Dimethyl sulfoxide (DMSO), MTT, propidium iodide (PI), Triton X-100, isopropanol, and puromycin were purchased from Sigma Chemical Co. (St. Louis, MO, USA). Bovine serum albumin and 2′,7′-dichlorofluorescein diacetate (DCFH-DA) were purchased from Beyotime Institute of Biotechnology (Jiangsu, China). The glucose, glutamine, and lactate assay kits were obtained from Mikebio (Chengdu, China). The total glutathione and ATP assay kits were purchased from Promega (Madison, WI) and Molecular Probes (Leiden, NL), respectively. The PicoPure RNA Isolation kit was obtained from Molecular Devices (Sunnyvale, CA). TRIzol, Lipofectamine^®^ 2000, and Superscript II RNase H-Reverse transcriptase were purchased from Invitrogen (Carlsbad, CA). The SsoFast^TM^ EvaGreen^®^ Supermix and the iScript^TM^ cDNA Synthesis kit were obtained from Bio-Rad Laboratories (Hercules, CA, USA).

Antibodies for GS and β-actin were purchased from Abcam (Cambridge, UK) and Cell Signaling Technology (Boston, US), respectively. All of the chemicals employed in this study were culture grade and of analytical purity.

### Cell lines

Cell lines used in this study were acquired from the American Type Culture Collection (Manassas, VA, USA). The gefitinib-resistant HCC827 cells (HCC827 GR) was a gift from Dr. Shengyong Yang. Apart from HEK293T cells, which were cultured in Dulbecco’s modified Eagle’s medium, all cells were cultured in RPMI 1640 media supplemented with 10% fetal bovine serum (FBS, Gibco, Auckland, N.Z.), 100 units/mL penicillin, and 100 units/mL streptomycin at 37 °C in a 5% CO_2_ humidified atmosphere.

Stable cell lines were created with lentiviral expression systems obtained from System Biosciences (SBI, Mountain View, CA, USA). In the first step to acquire *GLUL*-expressing A549 cells (A549-*GLUL*), the human *GLUL* cDNA sequence, which was obtained from Origene, CR204039 (Rockville, MD), was inserted into the pCDH vector to generate the overexpression vector. This vector was co-transfected with the packaging vectors psPAX and pMD2.G at a ratio of 2:2:1 into HEK293T cells using Lipofectamine^®^ 2000 3 days before the virus-containing media were harvested. The viruses were used to infect A549 cells. Two days later, 1.5 mg/mL puromycin was added to select positive cells.

Lentiviral shRNAs targeting *GLUL* were purchased as part of the human GIPZ lentiviral shRNAmir target gene set from Open Biosystems (Lafayette, CO). The procedures used for lentivirus generation and transfection were the same as described above. Multiple shRNA clones for the *GLUL* gene were used in the experiments.

### Cell viability assays

The cell viability of gefitinib-treated cancer cells was determined using the MTT assay. Briefly, cells (3 × 10^3^–5 × 10^3^ cells/well) were seeded on 96-well plates. After a 24-h incubation, cells were treated with various concentrations of gefitinib for 72 h. Then, 20 µL of a 5 mg/mL MTT solution was added, and the plates were incubated for an additional 2–4 h at 37 °C. The medium was subsequently discarded, and 150 µL of DMSO were added to dissolve the formazan crystals. The optical density was measured at 570 nm (OD_570_) using a multimode plate reader (Varioskan Flash, Thermo Fisher Scientific, Inc), and the IC_50_ values were calculated.

### Apoptosis analysis using flow cytometry

Cell apoptosis was analyzed using a previously described method^36^, with slight modifications. Briefly, cells were seeded on six-well plates. After 24 h of growth, change medium containing gefitinib for an additional 24 h and then harvested. Cells were washed twice with ice-cold phosphate-buffered saline (PBS) and incubated with 50 µg/mL PI containing 0.1% Triton X-100 for 30 min in the dark. Fluorescence was recorded using flow cytometry (FCM; BD FACSCalibur, USA), with excitation at 488 nm and emission at 617 nm.

### Metabolite extraction

After separately exposing A549 and PC-9 cells to 20 µmol/L and 20 nmol/L gefitinib, respectively, for 24 h, cells were harvested by centrifugation, and each sample contained at least 5 × 10^7^ cells. Then, cells were washed twice with precooled PBS, immediately frozen in liquid nitrogen, and stored at −80 °C. Intracellular metabolites were extracted using a modified Bligh-Dyer procedure^37^. Briefly, cell pellets were ultrasonically disrupted after the addition of bi-distilled water containing methanol (16 mL/g of cell pellet). After complete lysis, 8 mL of chloroform was added per gram of cell pellet, and the samples were homogenized again. Then, the suspension was mixed with another 8 mL/g chloroform and bi-distilled water. After a 30-min incubation on ice, the samples were centrifuged at 4000 × *g* for 30 min. The upper phase (aqueous phase) was collected and dried overnight in a centrifugal vacuum concentrator. The dried polar extracts were re-dissolved with 580 µL D_2_O containing 0.01 mg/mL TSP and 30 µmol/L PBS, pH 7.4. After centrifugation at 12 000 × *g* for 5 min, the supernatant was transferred into a 5-mm NMR tube and analyzed by ^1^H-NMR spectroscopy.

### ^1^H-NMR measurements

All samples were detected with ^1^H-NMR spectroscopy at 600.13 MHz using a Bruker-Av II 600 spectrometer (Bruker Co., Germany) at 300 K. Water signals were suppressed by pre-saturation. A 1D spectrum was acquired using a standard Carr-Purcell-Meiboom-Gill pulse sequence to suppress the water signal, with a relaxation delay of 5 s. Typically, 64 free induction decays (FIDs) were collected into 64 K data points over a spectral width of 12 335.5 Hz, with an acquisition time of 2.66 s and a total pulse recycle delay of 7.66 s. The FIDs were weighted by an exponential function with a 20.3-Hz line-broadening factor prior to Fourier transformation. Spectra were phased, baseline-corrected, and referenced to the TSP singlet at *δ* 0 ppm^38^.

### ^1^H-NMR spectral data reduction and pattern recognition analysis

The spectral region from *δ*9.5–0.5 for each sample was manually reduced to 4,384 integral segments of 0.002-ppm equal lengths using MestRe-c2.3 software (http://mestre-c-lite.findmysoft.com/download/). The area under the spectrum was then calculated for each segmented region and expressed as an integral value. The segments of *δ* 4.9–4.7 ppm in the spectra were excluded to eliminate the imperfect water resonance. The integrated data were normalized to the total sum of the spectrum prior to multivariate statistical analysis to yield the same total integration value for each spectrum.

Normalized NMR data were imported into SIMCA-P 11.0 (Umetrics, Sweden) for the multivariate statistical analysis. Variables were mean-centered and pareto-scaled for the PCA and PLS-DA. The PLS-DA models were cross-validated by permutation tests (permutation number = 200)^39,40^. The model coefficients locate the NMR variables associated with a specific intervention as *y* variables. The model coefficients were then back-calculated from the coefficients by incorporating the weight of the variables to enhance the interpretability of the model; in the coefficient plot, the intensity corresponds to the mean-centered model (variance), and the color scale derives from the unit variance-scaled model (correlation). Thus, biochemical components responsible for the differences between samples detected in the scores plot are extracted from the corresponding loadings, with the weight of the variable contributing to the discrimination. The coefficient plots were generated with MATLAB scripts with some in-house modifications and were color-coded with the absolute value of coefficients (*r*). In our study, the correlation coefficient of |*r*| > 0.637 was used as the cutoff value for statistical significance based on the discrimination significance at *p* < 0.05, which was determined using the test for the significance of Pearson’s product-moment correlation coefficient.

Class-specific metabolomics patterns were visualized using heat maps based on the acquired discriminating chemical shifts. The endogenous metabolites corresponding to the chemical shifts were assigned based on previous publications and the Human Metabolome Database (http://www.hmdb.ca/), a web-based bioinformatic/cheminformatic resource with detailed information about metabolites and metabolic enzymes.

### Analysis of glucose, glutamine, and lactate levels

The glucose, glutamine, and lactate levels were measured using commercially kit (Hitachi 7020 Automatic Analyzer, Japan). Cells were cultured in fresh media with or without gefitinib for 24 h, and metabolite concentrations in the media were measured according to the manufacturer’s protocol. The metabolite level was normalized to the total protein in each sample.

### Measurement of GSH levels

The intracellular GSH level was detected using the GSH-Glo glutathione assay kit (Promega Co., Madison, WI, USA) according to the manufacturer’s protocol. Briefly, cells were treated with gefitinib for 24 h, harvested by centrifugation, and washed twice with PBS to measure the total GSH levels (reduced GSH levels plus oxidized glutathione disulfide levels). The cell pellets were re-suspended in 50 μL of PBS, and 50 μL of 2× GSH-Glo reagent and 500 μM Tris (2-carboxyethyl) phosphine, a reducing agent, were added. In the presence of GSH, the luciferin derivative was converted into luciferin by glutathione *S*-transferases. After a 30-min incubation at room temperature, 100 μL of luciferin detection reagent were added to the sample and incubated an additional 15 min. The luminescence was measured using a 20/20 luminometer (Turner Designs). GSH concentrations were calculated from the standard curve acquired from known concentrations of GSH.

### Intracellular ROS assay

Intracellular ROS was assessed using non-fluorescent DCFH-DA^41^. ROS oxidize DCFH-DA to the fluorescent compound 2,7-dichlorofluorescein (DCF). DCF formation was used as a qualitative marker of cellular oxidative stress. Briefly, after treatment with 0, 0.1, 1, or 10 µM gefitinib for 72 h, cells were washed twice with PBS, and then incubated with 10 μM DCFH-DA in RPMI 1640 for 30 min in the dark at 37 °C. The media was removed, and cells were washed with PBS, detached with trypsin, and washed twice more with PBS. After centrifugation, the pellet was re-suspended in PBS, and the intracellular ROS level was detected by measuring the resulting fluorescent signal using FCM, with excitation and emission spectra of 488 and 529 nm.

### ATP measurement

ATP levels in cultured cells were measured as previously described^42^. Briefly, cells were seeded on 96-well plates. After 24 h of growth, media containing different concentrations of gefitinib were replaced, and cells were incubated for an additional 72 h. The culture medium was discarded, and cells were washed twice with ice-cold PBS. Then, the cells were lysed with ice-cold ATP buffer (20 mM Tris (pH 7.5), 0.5% Nonidet P-40, 25 mM NaCl, and 2.5 mM EDTA) on ice for 10 min. After centrifuging the cells at 13 000 × *g* for 30 min, the supernatant was collected and used to measure the protein and ATP concentrations. The cellular ATP levels were detected using an ATP determination kit (#A22066, Thermo Fisher Scientific, Inc., Waltham, MA, USA). For this assay, 10 µL of cell supernatant (0.5 µg of total protein) were mixed with 90 µL of reaction solution, and the luminescence was measured at 560 nm using a multimode plate reader (Varioskan Flash, Thermo Fisher Scientific, Inc.). The ATP levels were measured in each sample in triplicate. The ATP concentration was calculated from the standard curve and normalized to the total protein concentration.

### Microarray analysis

After treatment with gefitinib for 24 h, A549 and PC-9 cells (15 000–20 000 cells/sample) were harvested and washed twice with ice-cold PBS. RNA was extracted using the PicoPure RNA Isolation kit, and cDNAs were prepared using Superscript II RNase H-Reverse transcriptase. Gene expression profiling was performed using SmartArrays^TM^ (CapitalBio Corp., Beijing, China). The fluorescence intensities of the microarray spots were measured using a laser double-channel LuxScan 10KA scanner (CapitalBio Corp., Beijing, China). Image analysis was performed using GenePix Pro 4.0 software (Axon Instruments, Inc., Foster City, CA, USA). Then, Lowess normalization was applied to the primary data. After normalization, the ratio was calculated.

### qRT-PCR analysis

RNA was extracted from the experimental cells using Trizol and chloroform according to the manufacturer’s protocol. Isopropanol was used to precipitate the RNA, and the final RNA sample was dissolved in RNase-free water; cDNAs were prepared using the iScriptTM cDNA Synthesis kit. qRT-PCR was performed on cDNA samples using the Quantitative SsoFast TM EvaGreen^®^ Supermix and the CFX96™ Real-Time System or iQ™ SybrGreen Supermix CFX96 Real-time system (Bio-Rad).

The following primers were used in this study:

Human GGCT forward, 5′-CCAAGGCAAAACAAGTCAAAC-3′;

Human GGCT reverse, 5′-ACTACTCCCCACACTTCATCG-3′;

Human GLS forward, 5′-ACTGGCTAATGGTGGTTTCTG-3′;

Human GLS reverse, 5′-TTCCCCACAACTAAAAGAATGC-3′;

Human *GLUL* forward, 5′-GGGAGGAGAATGGTCTGAAGT-3′;

Human *GLUL* reverse, 5′-GCTACACCAGCAGAAAAGTCG-3′;

Human MGST2 forward, 5′-CTGCTGGCTGCTGTCTCTAA-3′;

Human MGST2 reverse, 5′-TGTTGTGCCCGAAATACTCTC-3′;

Human NADSYN1 forward, 5′-GCTCTCGGTCTATGGGAAACT-3′;

Human NADSYN1 reverse, 5′-GAGCGTGGTCATCTTGTGTCT-3′;

Human ODC1 forward, 5′-TGAAGGTTTTACTGCCAAGGA-3′;

Human ODC1 reverse, 5′-TCTTCACGATGGCTTTGCTAT-3′;

Human RRM1 forward, 5′-TCCTGATGACCTGAAGCAACT-3′;

Human RRM1 reverse, 5′-CTCAGCAATGTGGATGTTCAA-3′;

Human RRM2 forward, 5′-GATTGGGGACAAAGAGGCTAC-3′;

Human RRM2 reverse, 5′-AGGCATCAGTCCTCGTTTCTT-3′.

### Western blotting analysis

Cells were harvested and washed twice with ice-cold PBS. Then, radioimmunoprecipitation assay lysis buffer (50 mM Tris, 150 mM NaCl, 0.1% SDS, 0.5% sodium deoxycholate, 1% Triton X-100, 1 mM phenylmethylsulfonyl fluoride, 10 mM sodium azide, 10 mM sodium ascorbate, and 5 mM trolox) was added to lyse the cells on ice for 10 min. The lysates were briefly sonicated and placed on ice prior to centrifugation at 13 000 rpm for 10 min. Proteins were separated on 12% SDS-PAGE gels and probed using the indicated antibodies. The primary antibodies used in this study were anti-GS (Abcam, ab176562, 1:500) and anti-β-actin (CST, #4970, 1:500). After an incubation with horseradish peroxidase-conjugated secondary antibodies (Promega), the bands were visualized using the enhanced chemiluminescence method.

### Migration and invasion assay

Cell motility and invasion were examined using scratch wound-healing assays^43^ and Transwell migration^44^, respectively. For the scratch wound-healing assays, a cell monolayer was scratched with a 200-μL pipette tip after a 24-h incubation with media containing 0.5% serum. The scratch resulted in a cell-free gap or “wound” of approximately 1.0 mm between two adjoining areas. Immediately after wounding, growth media (containing 10% FBS) with or without gefitinib were added, and images were captured of the intersections of the linear wound with an Olympus digital camera. The area covered by cells that had migrated into the wound was determined using an area measurement program (SpotSoftware, Version 4.6, Diagnostic Instruments). Experiments were performed in at least seven separate dishes and the results were averaged.

Cell invasion was detected by assessing the cell migration rate through an artificial basement membrane in a modified Boyden chamber (Corning Costar, Fisher Scientific). The membrane consisted of polycarbonate (0.4-µm pore diameter) and was coated with Matrigel (BD Biosciences) diluted in serum-free RPMI 1640 on ice. Cells re-suspended in culture medium were seeded into the upper well of the chamber (100 μL), and the lower well was filled to the top (approximately 600 μL) with RPMI 1640 medium containing 10% FBS. Cells were incubated for 24 h. The cells in the upper well that did not migrate were scraped off, and the cells that migrated to the lower side of the membrane in the upper well were stained with crystal violet. Invading cells were observed under a microscope.

### Statistical analysis

The data are expressed as the mean ± standard deviation. All analyses were performed using the Statistical Package for the Social Sciences software version 13.0 (SPSS Inc., Chicago, IL). Standard error of mean is represented as error bars, and statistical significance is indicated. A value of *p* < 0.05 was considered significant.

## Electronic supplementary material

Supplemental information

Figure S1

Figure S2

Figure S3

Table S1

Table S2

Table S3

Table S4

Table S5

Table S6
