# Supplementary material for: Increased glutamine anabolism sensitizes non-small cell lung cancer to gefitinib treatment
Source: Cell Death Discov. 2018 Aug 9;4:84. doi: 10.1038/s41420-018-0086-x (PMC6085389; doi:10.1038/s41420-018-0086-x)
Supplement: Supplementary file 1 — Supplemental information [file 41420_2018_86_MOESM1_ESM.docx]

**Supplemental information**

**Supplemental figure legends**

**Figure S1.** The percentage of dead A549 and PC-9 cells was analyzed by FACS following a 24-h treatment with 20 µM and 20 nM gefitinib, respectively.

**Figure S2.** Changes in the ratio of the *GLS*/*GLUL* mRNAs in A549 and PC-9 cells that were treated as described in Supplementary 2. The levels of the *GLS* and *GLUL* mRNAs were first normalized to the *GAPDH* mRNA levels. The data represent the mean ± SEM of 3 independent experiments. *** *p* < 0.001, 2-tailed Student’s *t*-test.

**Figure S3.** qRT-PCR was used to quantify the changes in *GLS* mRNA expression in A549 and PC-9 cells after a 48-h treatment with 20 µM or 20 nM gefitinib, respectively. The bars shown are normalized to the GAPDH control and represent the mean ± SD of triplicate samples.
