# Supplementary figures and images for: Increased glutamine anabolism sensitizes non-small cell lung cancer to gefitinib treatment
Source: Cell Death Discov. 2018 Aug 9;4:84. doi: 10.1038/s41420-018-0086-x (PMC6085389; doi:10.1038/s41420-018-0086-x)

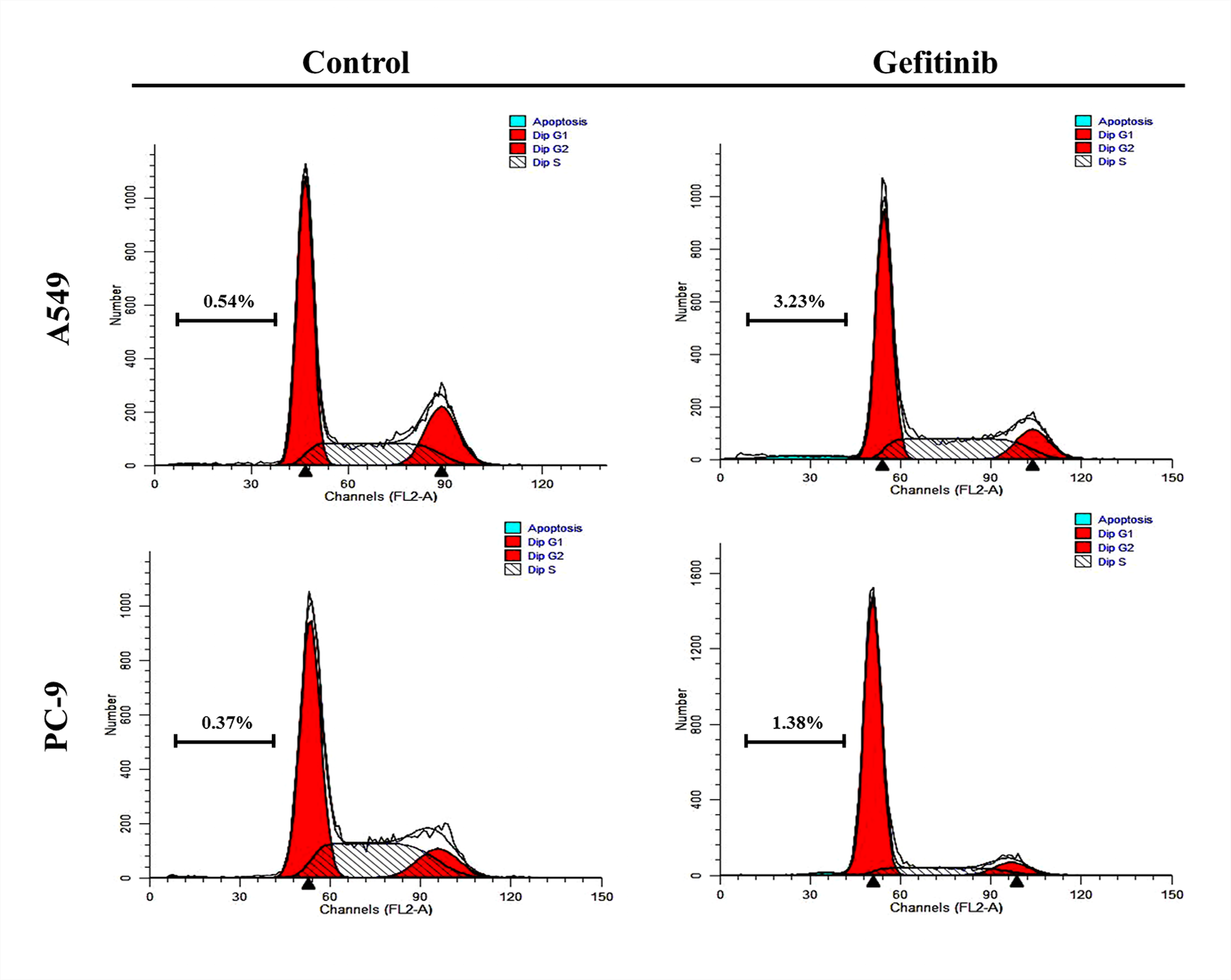

Supplement: Supplementary file 2 — Figure S1 [file 41420_2018_86_MOESM2_ESM.tif]

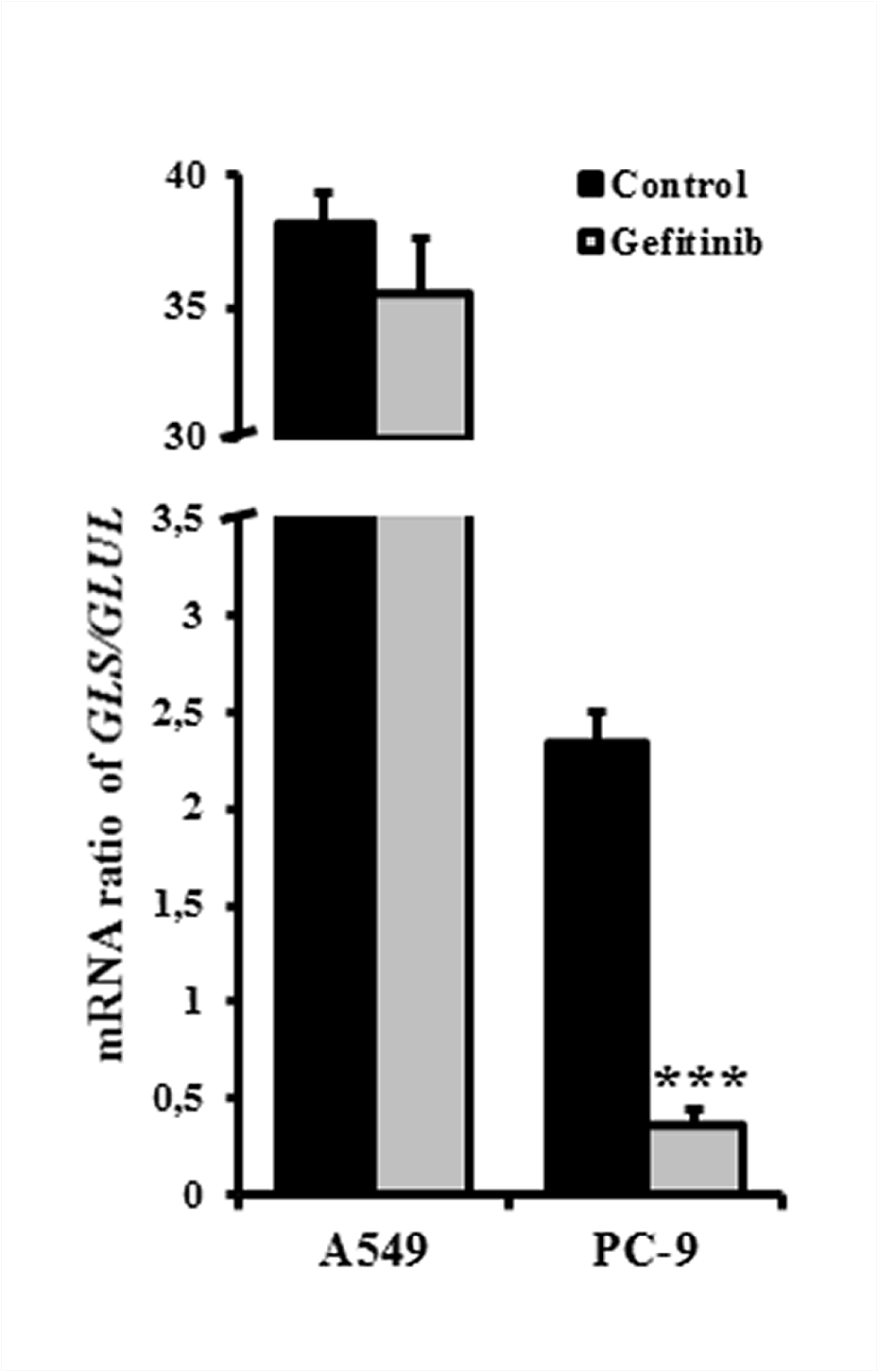

Supplement: Supplementary file 3 — Figure S2 [file 41420_2018_86_MOESM3_ESM.tif]

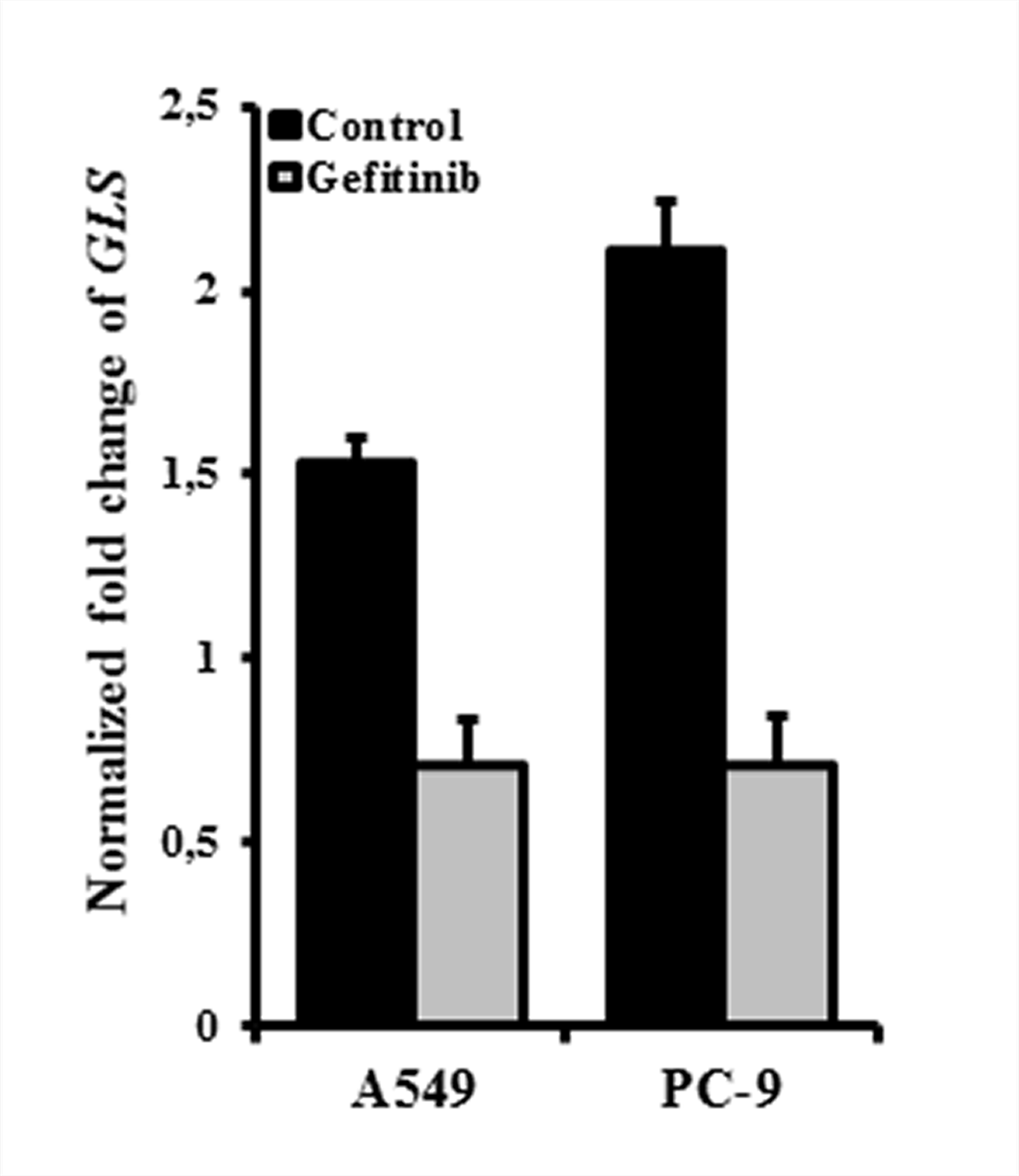

Supplement: Supplementary file 4 — Figure S3 [file 41420_2018_86_MOESM4_ESM.tif]
